# Defining Human Differences in Biomedicine

**DOI:** 10.1371/journal.pmed.0040288

**Published:** 2007-09-25

**Authors:** Maggie Brown, The PLoS Medicine Editors

A study is published reporting that a new drug works better in black people than in whites. Is this an informative study or is it based on archaic, incorrect, even harmful notions of human difference?

*Homo sapiens* has been called the species that names. An extensive literature reflects millennia of concern over what we humans call ourselves and others. All life sciences are now grappling further with how to categorize and study the nearly infinite polymorphisms within and among “species” as awareness grows that the species concept itself is inadequate. Human medicine, however, is in a unique position in that not only must it confront these problems of categorization that plague all life sciences, but this effort occurs in a complex sociopolitical context. As Smart and colleagues recently said, “One reason that race and ethnicity are difficult concepts to operationalize or examine in scientific research is that they have meaning and usage that exists beyond the domain of scientific control” [1].

## Dangers of Designations

In this issue of *PLoS Medicine*, Braun et al. [2] discuss some of the difficulties and dangers that go along with racial and ethnic designations in the clinic and in biomedical research. They propose that a clear consensus needs to be reached about exactly how to use race and ethnicity as variables and as designations and when it is valid to use them. They state further that while race may be used legitimately as a “descriptive” quality, it should not be used as a “biological attribution.” In other words, race as a social construct may result in differences in treatment that affect health outcomes, but such descriptive use does not imply that race can be used as a proxy for biological difference. In a commentary on the Fausto-Sterling essay, Ellison et al. [3] agree that standards of definition need to be improved, but caution that potentially important data may be at stake: citing the example of variance in histocompatibility antigen markers as important in transplantation research, they argue that variation in the frequency of genotypic markers among racial and ethnic groups should not be unilaterally discarded simply because of the risk that such information may be misused. They add that while international consensus on improved categorization is important, any guidelines deriving from such consensus will need to be made flexible: “such categories cannot and should not be standardised for use in all scientific, social, and clinical contexts.” This proposal echoes a *Nature Genetics* editorial that argued for inclusion of multiple types of information (e.g., “ancestral and environmental”) so that the data can be “grouped flexibly to serve the needs of medical geneticists, epidemiologists, and biological anthropologists” [4]. To complicate matters, a constraint facing many researchers is the requirement that US National Institutes of Health grantees in clinical research collect information on race that follows the current US census designations [5,6].

With this range of opinions, lack of unity and authority, and constraining rule from the largest granting agency in the US, the debate on whether and how to use race and ethnicity in biomedical research and its reporting seems almost intractable.

In journal publishing particularly, the need for clarity and guidance on how to define and use race and ethnicity is increasing, especially over the last decade or so as the numbers of studies in two specific areas increase: genetic associations and studies of health inequities [7]. However, one analysis of a sample of 72 articles on cardiovascular disease reported that only 39 (55%) of articles referring to race/ethnicity described in detail how race/ethnicity was determined [8]. Further, a 2004 study of 120 genetics and heredity journals found that only two included instructions to authors regarding race and ethnicity classifications [9]. Yet over half of the journals had published articles using racial or ethnic categories in the previous ten-year period. Even among journals with specific guidelines, adherence tends to be poor [7].

## Solutions Are Possible

What can journals do? Should designations of race and ethnicity be left to an author's discretion, since, after all, authors know the most about their own studies? Some editors have adopted such an approach, arguing that the author and referees are best equipped to offer expert guidance on the matter [1,9]. Other journals, instead of creating specific instructions for authors, have published position papers that seek to persuade, rather than force, authors to apply more consistent, rigorous standards of terminology and science (e.g., [10,11]). Kaplan and Bennett [11], for example, suggest that, whenever possible, studies of racial differences should include analyses to control or adjust for other variables, such as socioeconomic status, nutrition, environmental exposures, etc. (see also [12]). In 2004 *Nature Genetics* devoted an entire special issue (“Genetics for the Human Race”) to a discussion of how to conceptualize, define, and study human racial and genetic differences (content freely available at http://www.nature.com/ng/journal/v36/n11s/index.html). As noted above, a few journals have added instructions or guidance to their author guidelines (e.g., *Journal of the American Medical Association*, http://jama.ama-assn.org/misc/ifora.dtl#ReportingRaceEthnicity; *Canadian Medical Association Journal*, http://www.cmaj.ca/authors/policies.shtml; *Genetics in Medicine*, http://edmgr.ovid.com/gim/accounts/ifauth.htm).

However helpful and forward-thinking these individual journal responses are, community consensus would be more useful and authoritative. General scientific or medical editorial style guides have added useful detail and explanations to their chapters on terminology with each new edition [13,14]. A well-known example of community adoption of guidelines is of those produced by the International Committee of Medical Journal Editors (ICMJE, http://www.icmje.org/). Over 600 journals (*PLoS Medicine* among them) have agreed to follow their *Uniform Requirements for Manuscripts Submitted to Biomedical Journals* [15]. Regarding race and ethnicity (and by implication other categories), the guidelines state, “The guiding principle should be clarity about how and why a study was done in a particular way. When authors use variables such as race or ethnicity, they should define how they measured the variables and justify their relevance” (section IV.A.6.a., Selection and Description of Participants [15]). Some authors feel, however, that while they provide a good foundation, these guidelines are not detailed or comprehensive enough [1,9,11].

A more detailed community consensus would provide the benefits of uniformity and legitimacy for both authors and editors. In addition to ICMJE, AMA (American Medical Association, http://www.ama-assn.org/), and CSE (Council for Science Editors, http://www.councilscienceeditors.org/), other organizations that might help develop such international consensus include WAME (World Association of Medical Editors, http://www.wame.org/) and EASE (European Association of Science Editors, http://www.ease.org.uk/).

## More Than Race and Ethnicity

Although race and ethnicity as contentious variables in research and clinical medicine are the most discussed in the literature, they are not the only possible sources of incorrect generalizations and possibly harmful bias. Others are sex/gender, age, sexual orientation, disease/disability, religion, socioeconomic status, and many more. For example, the AMA *Manual of Style* (10th edition, section 11.10 [13]) and the CSE manual (7th edition, section 7.5 [14]) offer advice on inclusive language in the areas of race/ethnicity, age, disease/disabilities, religion, and sexual orientation, emphasizing in part that terminology should be nonstigmatizing and reflect the preferred designations of groups or individuals. In all of these areas humans have been subject to stereotyping and discrimination; thus a critical examination of all the names we call ourselves and others is warranted, and at least general guidelines should be developed for these areas, although consensus may take time.

## Who Is Responsible?

Whose responsibility should it be to ensure that race, ethnicity, and other human variables are described appropriately? Authors and referees, who know their research and fields better than most journal editors do? Or editors, whose mandate it is to uphold the scientific and ethical quality of their journals? The answer is both, working collaboratively.

Ideally, authors would design their studies, including the reporting of studies, to the highest standards available at the time. Because those standards keep changing, however, editors can't make authors go back in time and redesign a decades-long prospective study to meet contemporary editorial standards. Editors should insist, however, that authors of studies based on different human categories should make their methods of categorizing human populations transparent, justify their study design, and control for confounding variables. And copyeditors can help by being well informed about current trends in usage, and should query authors about what, exactly, is meant by “black” or “homosexual” or “poor” or “elderly” when these designations are not clear, and recommend changing potentially offensive labels to more acceptable terminology (e.g., “diabetics” to “participants with diabetes”).

The biomedical landscape is becoming rapidly more complex. Burgeoning human genome and epidemiologic data, racially defined pharmaceutical treatments, and increased public concern over scientific research ethics combine to force everyone involved to confront the problems of defining and studying the causes and consequences of human differences. It is clear that although much can be done to improve clarity of reporting, race, ethnicity, religion, sex, sexual orientation, age, disease, disability, weight, and any of the multitude of other ways of categorizing humans are here to stay.
